# A bibliometric analysis of targeted therapy cardiotoxicity research in cancer patients (2004–2024)

**DOI:** 10.3389/fmed.2025.1593178

**Published:** 2025-06-26

**Authors:** Guoming Chen, Guang Chen, Huiping Zhou, Qingyi Yang, Yifan Shang, Rui Qin, Yingyue Hou, Cheng Zhang, Jiarui Lin, Xuan Ye, Ning Wang, Yibin Feng

**Affiliations:** ^1^School of Chinese Medicine, Li Ka Shing Faculty of Medicine, The University of Hong Kong, Hong Kong, Hong Kong SAR, China; ^2^Foshan Clinical Medical College, Guangzhou University of Chinese Medicine, Foshan, China; ^3^Clinical Medical College of Acupuncture-Moxibustion and Rehabilitation, Guangzhou University of Chinese Medicine, Guangzhou, China; ^4^College of Traditional Chinese Medicine, Guangzhou University of Chinese Medicine, Guangzhou, China; ^5^First Clinical Medical College, Guangzhou University of Chinese Medicine, Guangzhou, China; ^6^School of Information Engineering, Guizhou University of Traditional Chinese Medicine, Guiyang, China

**Keywords:** targeted therapy, cardiotoxicity, bibliometrics, cancer, VOSviewer, CiteSpace

## Abstract

**Background:**

The management of long-term cardiotoxicity has become increasingly challenging despite the growing utilization of targeted therapies to enhance progression-free and overall survival rates. Although there is a proliferation of literature on the incidence and mechanisms of cardiotoxicity induced by targeted therapies, no comprehensive analysis of the publication landscape has addressed the unmet medical needs in this area. This study aimed to characterize global research trends, map collaborative networks, and highlight unresolved issues in cardiotoxicity management to fill the gaps in this field and inform future research.

**Method:**

This study conducted a bibliometric analysis of articles concerning targeted therapy-induced cardiotoxicity published between 2004 and 2024 from the Web of Science (WOS) database using VOSviewer and CiteSpace. A total of 1,054 publications from 71 countries/regions and 2,058 research institutions were examined.

**Result:**

The number of publications has shown an average annual increase of 50 articles from 2004 to 2024. Key research topics in targeted therapy cardiotoxicity encompass breast cancer, heart failure, and drug delivery. The most cited publication is a guideline titled “Prevention and Monitoring of Cardiac Dysfunction in Survivors of Adult Cancers: American Society of Clinical Oncology Clinical Practice Guideline.” These results indicate a rising trend in research on tumor-targeted therapy cardiotoxicity over the past two decades. Recent research trends and future directions primarily focus on two key areas: the development of novel nanocarriers aims to enhance therapeutic efficacy while reducing cardiac toxicity, and the exploration of mechanisms underlying cardiac injury caused by targeted therapeutic drugs is crucial, along with the investigates drug interventions to counter these mechanisms or the application of alternative techniques for the prevention, alleviation, or treatment of cardiac injury.

**Conclusion:**

This study provides a comprehensive overview of targeted therapy-induced cardiotoxicity research from 2004 to 2024. By identifying key research priorities, this analysis addresses critical gaps in current knowledge. Future endeavors should prioritize translational innovations and multidisciplinary clinical frameworks to enhance therapeutic safety.

## 1 Introduction

Cancer is a major public health issue worldwide. The incidence of cancer has been increasing over the past two decades. Each year, over 1 million people are diagnosed with cancer, while many of them have the possibility of facing the risk of treatment-related complications (1). Currently, the main treatment modalities for cancer include surgery, radiation therapy, chemotherapy, immunotherapy, and molecular-targeted therapy. Among these, molecular targeted therapy (MTT) stands out as a promising strategy that targets specific molecules to impede cancer growth, progression, and metastasis (2). Initially used as palliative care for advanced solid tumors, molecular targeted therapy (MTT) has evolved into a curative option for early-stage disease (3). Notably, it is important to note that cardiac toxicity is a common adverse reaction associated with molecular targeted therapy, and major drugs associated with cardiac toxicity include small molecule tyrosine kinase inhibitors and other molecular targeted drugs, including monoclonal antibodies (4). Drug-induced cardiac toxicity manifests in various impacts on cardiac structure and function, such as hypotension, hypertension, left ventricular dysfunction, prolonged QT interval, myocardial ischemia, and thromboembolism (5).

Over the past decade, research on cardiac toxicity induced by targeted cancer therapies has made significant progress, but a comprehensive summary of the research content and advancements is still lacking. The purpose of this study is to systematically analyze the global research landscape of cardiotoxicity induced by targeted therapy through bibliometric methods, with the aim of identifying key research hotspots, collaborative networks, and evolutionary trends. By identifying the major contributing countries, institutions, and authors in this field, the core research themes, the unresolved challenges in cardiotoxicity management, and potential future research directions, this study seeks to fill existing gaps in understanding, provide a theoretical foundation for future research, and offer practical guidance for clinical applications and interdisciplinary collaboration.

Bibliometrics, a quantitative analysis employing mathematical and statistical methods to evaluate research trends, serves as a crucial tool in this endeavor (6). Presently, scientific bibliometric analyses primarily rely on tools such as CiteSpace, VOSviewer, and HistCite (7), providing researchers with a comprehensive overview of fundamental data and dynamic trends. This aids in assessing existing issues and plays a pivotal role in predicting future research directions (8).

In this study, we conducted a bibliometric analysis to assess the research literature on cardiac toxicity of targeted cancer therapies from 2004 to 2024. We described the research status in this field and identified new research directions. The study systematically organized and evaluated the involvement of countries, authors, and institutions in this research. Furthermore, through cluster analysis and visualization of keywords, subject terms, and cited references, we anticipated potential future directions, thereby laying a theoretical foundation for further exploration by scholars in this field.

## 2 Methods

**Figure 1:**
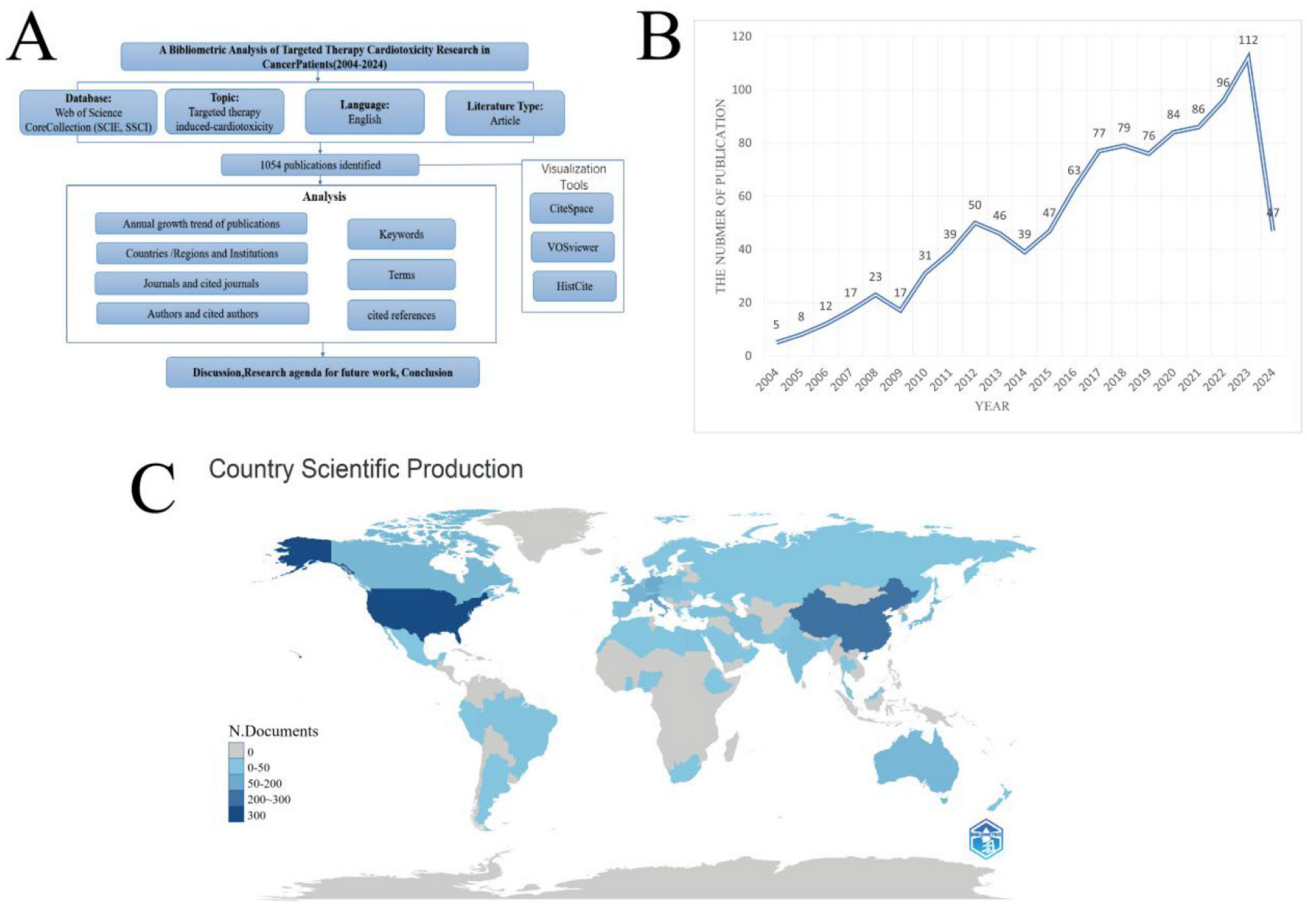
**(A)** Flowchart depicting the article selection process. **(B)** The line chart of publication growth in Cardiotoxicity of Targeted Cancer Therapies. **(C)** Comparative map of the cumulative number of papers published in each country. The darker the color, the more posts there are.

Clarivate Analytics' Web of Science (WOS) core database is considered the best bibliometric analysis database (9), which is selected to perform the search. We searched for all articles associated with cardiotoxicity of targeted cancer therapies from 2004 to 2024 on August 25, 2024, and use the following search formula: TS = (Neoplasms OR Tumor OR Neoplasia OR Tumors OR Neoplasias OR Cancers OR Cancer OR Malignant Neoplasm OR Malignancies OR Neoplasm OR Malignancy OR Malignant Neoplasms OR Neoplasm, Malignant OR Benign Neoplasms OR Neoplasms, Malignant OR Benign Neoplasm OR Neoplasm, Benign OR Neoplasms, Benign) AND TS = (Molecular Targeted Therapy OR Targeted Therapy, Molecular OR Targeted Molecular Therapy OR Therapy, Molecular Targeted OR Molecular Targeted Therapies OR Molecular Therapy, Targeted OR Targeted Molecular Therapies OR Therapy, Targeted Molecular OR Targeted Therapy) AND TS = (Cardiotoxicities OR cardiotoxicity OR Cardiac Toxicity OR Cardiac Toxicities OR Toxicity, Cardiac). The criteria for including literature were as follows: (1) the database source was limited to Science Citation Index Expanded and Social Sciences Citation Index; (2) document types included article; and (3) written in English (Figure 1A).

VOSviewer (version 1.6.19) (10) was applied to visualize a collaborative network map of institutions, countries, authors, and terms. We used VOSviewer to generate a network of subject terms and density visualization, selecting the top 60%. CiteSpace (version 6.3.R1) (11) was utilized in this study for visualizing keyword co-occurrence, keywords clustering, keyword time zone, burst analysis of keywords, cited journals, and cited references. Because the threshold is cumulative, some recent keywords may not have reached a count of 20. Therefore, we included the top four high-frequency keywords for each year from 2016 to 2024 to supplement the temporal analysis. HistCite (version Pro 2.1) was used to analyze journal publication volume.

## 3 Results

By searching the WOS database, from 2004 to 2024, 1,054 articles on the cardiac toxicity of targeted molecular therapy for cancer were identified (Figure 1A). These articles were published by 8,052 authors from 71 countries or regions, 1930 institutions, and 460 journals. The annual publication trend is depicted in Figure 1B, revealing a peak of 112 publications in 2023 and a nadir of 17 publications in 2009. Overall, a discernible upward trajectory in publication numbers is evident over the years, with an average of 50 publications annually. Disparities in publication counts were observed across different geographical regions (Figure 1C).

### 3.1 Regions/institutions and countries of publications

**Figure 2:**
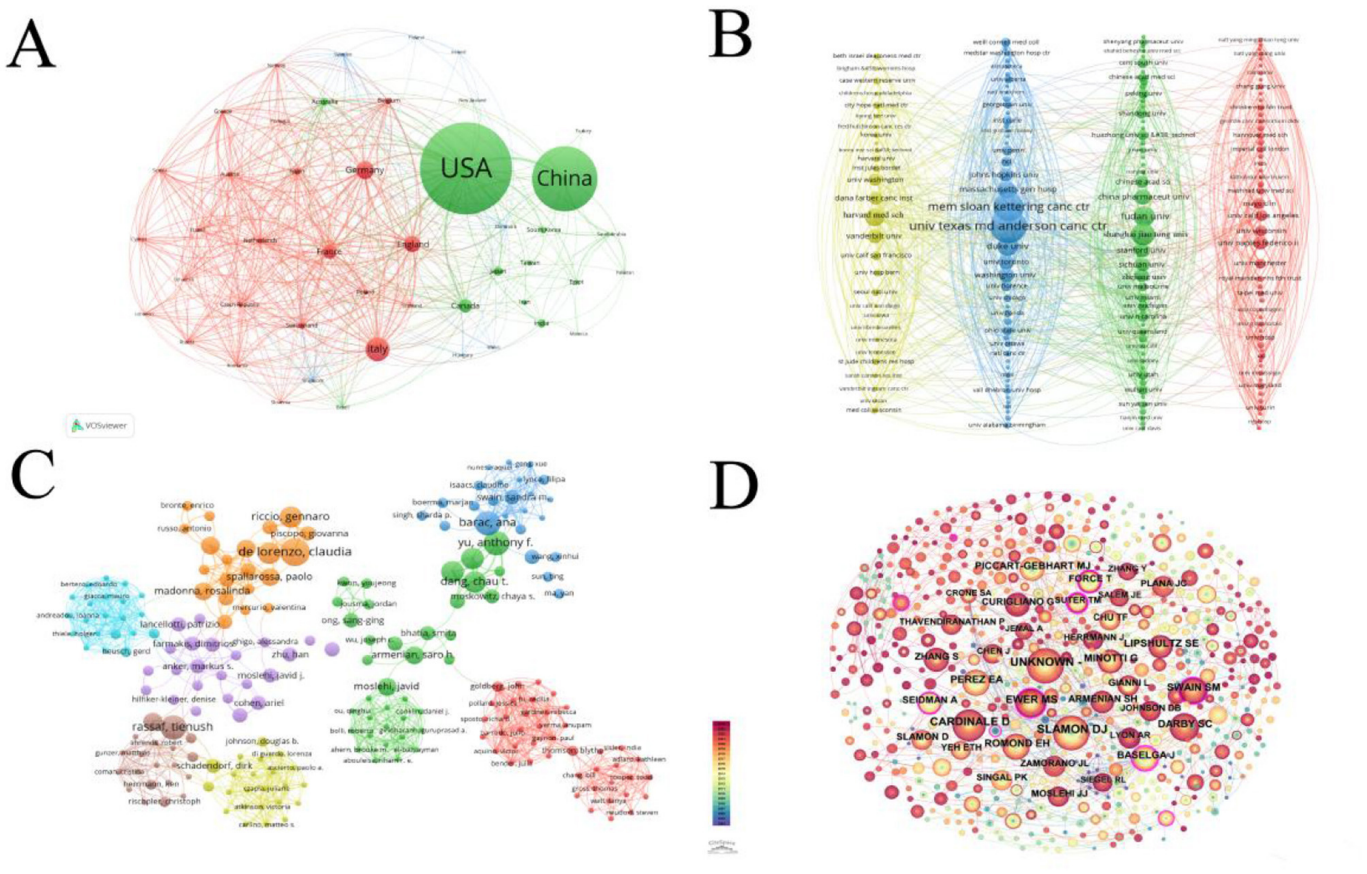
**(A)** This network selects the number of documents of a country that is more than 3. The more times these countries appear, the larger the dots and the darker the colors. The line connecting two adjacent circles represents the degree of correlation between the two circles. The more connections, the thicker the line. **(B)** This figure selects institutions with a publication volume >3. The more circles there are, the more articles the organization publishes. The thicker the line between two circles, the closer the connection between institutions. **(C)** Network map showing authors in Cardiotoxicity of Targeted Cancer Therapies. The graph consists of nodes representing authors, and the lines that connect the circles represent the relationships between authors who co-occur in their publications. The area of each circle represents the number of publications by the respective researcher. **(D)** Network map showing co-authors involved in Cardiotoxicity of Targeted Cancer Therapies. In the co-citation graph, authors are represented as nodes in the form of circles. Each node represents a core author, and the size of the node indicates the author's degree centrality, which is the number of other authors they are co-cited the thickness of the connecting lines represents the frequency of co-citation between two authors.

Figure 2A presents a network diagram illustrating the volume of research publications on targeted therapy for cardiotoxicity from 2004 to 2024 across various countries. A total of 1,054 articles were amassed from 71 countries, offering a global perspective on the publication output in this domain. The United States of America (USA) emerged as the most prolific publisher, contributing 372 publications, accounting for 35.3% of the total 1,054 articles. This was followed by China (264, 25.0%), Italy (98, 9.3%), Germany (74, 7.0%), and the United Kingdom (68, 6.5%). We described the research status in this field and identified new research directions. Notably, the USA (322) and the UK (244) exhibited the strongest collaborative power with other countries, representing 8.77 and 6.64% of the total collaborative strength (3,672), respectively. The global collaboration network, as depicted in Figure 2A, revealed a strong partnership between the USA and China, with China actively engaging in collaborations not only with the USA but also with France, Canada, England, Germany, and Italy.

Figure 2B displays a network diagram illustrating the collaboration strength among institutions, revealing the interconnections between them. The University of Texas MD Anderson Cancer Center emerged as the most prolific publisher, contributing 30 publications, accounting for 2.84% of the total 1,054 articles with 2,170 citations. The second-ranked institution is a private cancer center in the USA, Memorial Sloan Kettering Cancer Center (25 articles, 2.37%, with 1,710 citations). The top 10 highest-producing institutions are located in China and the USA, indicating close collaborations between them. In comparison to the top-ranking institution, the University of Texas MD Anderson Cancer Center, the second-ranked Memorial Sloan Kettering Cancer Center exhibited stronger collaborative power with other institutions, accounting for a total collaborative strength of 157, which represents 0.93% of the total collaborative strength (16,864) among all institutions. The University of Texas MD Anderson Cancer Center (129), on the other hand, contributed only 0.76% to the total collaborative strength.

### 3.2 Authors and journals

A total of 8,052 authors contributed to research on the cardiac toxicity of targeted molecular therapy for cancer. The top three authors with the highest number of publications are Claudia de Lorenzo (*n* = 7), Khalil Abnous (*n* = 6), and Thomas M. Suter (*n* = 6). A co-authorship network diagram, based on bibliometric analysis, is presented in Figure 2C, while a citation-based network diagram utilizing the most cited authors as nodes is depicted in Figure 2D.

**Figure 3:**
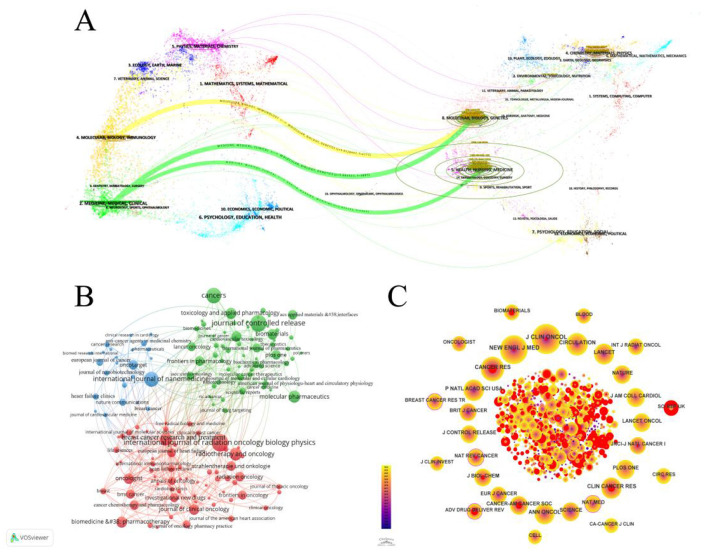
**(A)** The dual overlay of journals related to the cardiotoxicity of targeted cancer therapies is showcased. The citation graph is displayed on the left side, while the cited graph is shown on the right side. The curve depicts the verification line, with various reference relationships displayed in distinct colors. **(B)** Network map showing journals in Cardiotoxicity of Targeted Cancer Therapies. The graph consists of nodes representing journals, and the lines that connect the circles represent the relationships between journals who co-occur in their publications. The size of each circle indicates the number of articles published by that journals. **(C)** CiteSpace network visualization of journals related to cardiotoxicity of targeted cancer therapies. Nodes represent journals and lines representing co-occurrence between journals connect them. The node size is proportional to the frequency of the journals. The outermost layer of a node with a purple circle indicates high centrality of the represented keyword.

The analysis encompassed 460 journals (Figure 3B). The top four journals by article count were the International Journal of Radiation Oncology Biology Physics (20 articles, 1.89%), Journal of Controlled Release (19 articles, 1.80%), Cancer (17 articles, 1.61%), and International Journal of Nanomedicine (16 articles, 1.51%). Among the top 10 most prolific journals, Biomaterials boasted the highest impact factor (IF) at 12.8. Table 1 displays the top 10 journals in publications. TLCS displays the citation counts in the 460 journals which we analyzed. TGCS displays the number of citations in all journals. Performing visualization analysis of co-cited journals (Figure 3C), With 603 citations, the Journal of Clinical Oncology led in total mentions, followed by the New England Journal of Medicine (541 citations) and Cancer Research (390 citations). The Lancet, with an impact factor (IF) of 98.4, garnered 305 citations and emerged as the highest IF among the top 10 cited journals. The dual-map overlay of journals (Figure 3A) delineates three primary pathways, indicating that papers published in the “2 Medicine, Medical, Clinical” journal predominantly cite in the fields of “5 Health, Nursing, and Medicine” and “8 Molecular, Biology, Genetics.” Additionally, a significant frequency of citations from papers in the “4 Molecular, Biology, Immunology” field to journals focused on “8 Molecular, Biology, and Genetics” is notable.

**Table 1:** Top 10 Journals in the publications.

**Rank**	**Journal**	**Document**	**TLCS**	**TGCS**	**IF**
1	International Journal of Radiation Oncology Biology Physics	20 (4.35%)	16	977	6.4
2	Journal of Controlled Release	19 (4.13%)	15	1,187	10.5
3	Cancers	17 (3.70%)	0	117	4.5
4	International Journal of Nanomedicine	16 (3.48%)	7	428	6.6
5	Breast Cancer Research and Treatment	13 (2.83%)	25	382	3
6	Radiotherapy and Oncology	13 (2.83%)	12	542	4.9
7	Molecular Pharmaceutics	12 (2.61%)	2	465	4.5
8	Biomedicine and Pharmacotherapy	11 (2.39%)	1	201	6.9
9	Toxicology and Applied Pharmacology	11 (2.39%)	6	258	3.3
10	Biomaterials	10 (2.17%)	8	993	12.8

### 3.3 Top cited research in targeted therapy cardiotoxicity

**Figure 4:**
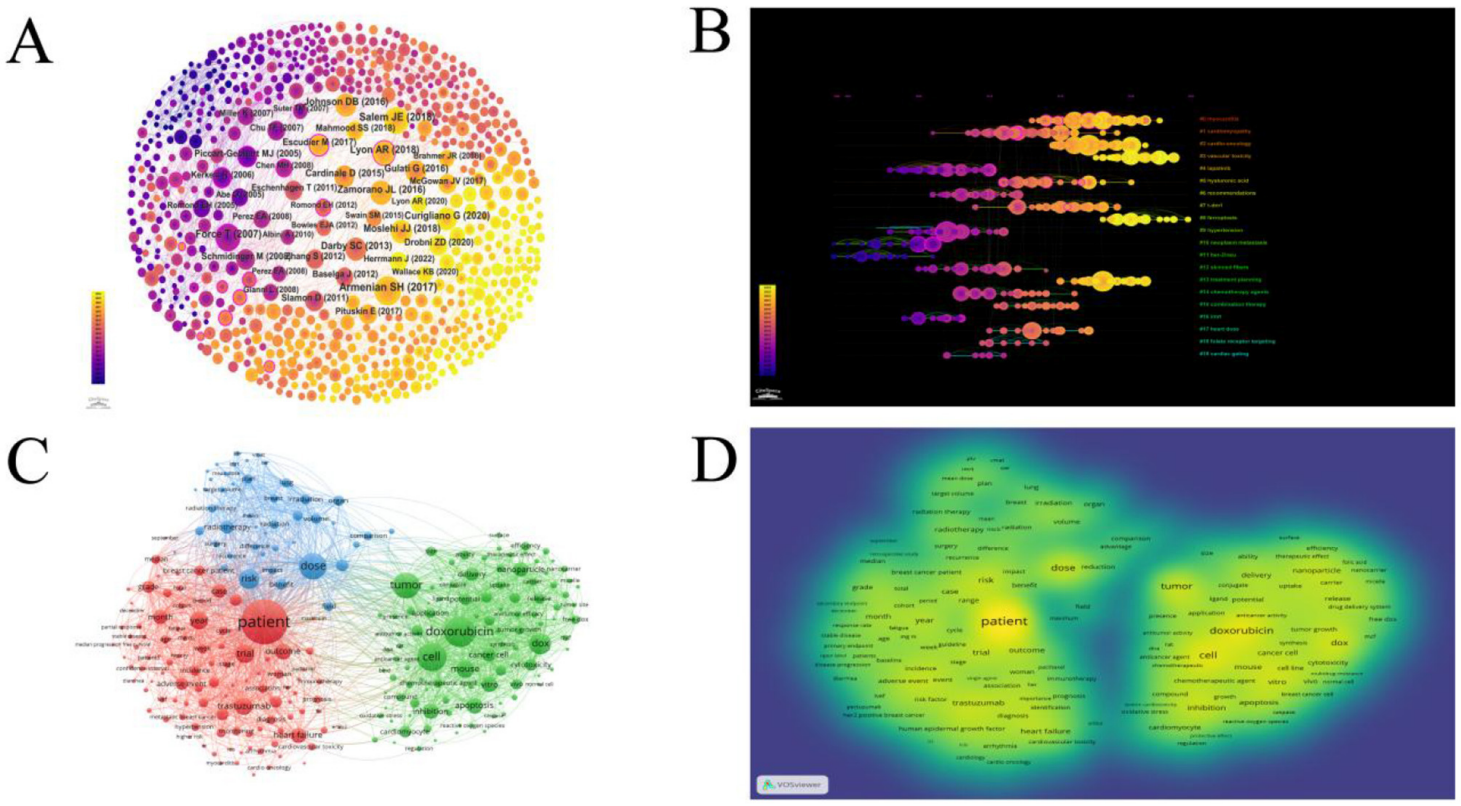
**(A)** Network map showing co-reference involved in Cardiotoxicity of Targeted Cancer Therapies. In the co-reference network, circles represent nodes, with each node representing a reference. The size of the node in the graph indicates the degree centrality of the reference, which refers to the number of other references that co-cite it. The thickness of the edges represents the frequency of co-citation between two references. **(B)** The timeline view of references about the research related to Cardiotoxicity of Targeted Cancer Therapies. Nodes of different colors on the same line represent distinct years. The straight lines at the same horizontal position represent the entirety of literature belonging to a particular cluster, with the cluster label positioned at the right end of the line. **(C)** The top 60% (226 Terms) were selected for generating network visualizations using VOSviewer. The subject terms are divided into three clusters, replaced by different colors or different color depths. The larger the circle, the more times the theme word appears. **(D)** The top 60% were selected for generating density visualizations using VOSviewer. Similarly, the darker the color, the more frequently it appears.

The article titled “Prevention and Monitoring of Cardiac Dysfunction in Survivors of Adult Cancers: American Society of Clinical Oncology Clinical Practice Guideline” holds the highest number of citations, totaling 29 citations, ranking first. The co-cited literature cluster analysis revealed a modularity *Q* value of 0.8945 and an average silhouette coefficient of 0.453 (Figure 4A). On the timeline, the closest clusters include “#8 ferroptosis,” “#3 vascular toxicity,” “#0 myocardities,” “#2 cardio-oncology,” “#13 treatment planning,” “#5 hyaluronic acid,” “#1 cardiomyopathy,” “#7 t-dm1,” “#17 heart dose,” “#14 chemotherapy agents,” and “#15 combination therapy” (Figure 4B).

### 3.4 Subject terms and clustering analysis

A total of 26,808 Mesh were extracted from 1,054 articles. Among these, 417 Mesh appeared more than 15 times. The top 250 Mesh were selected for generating network and density visualizations using VOSviewer (Figure 4C). In the Mesh clustering network, there were 250 nodes connected by 17,632 lines. Three main clusters can be observed, dominated by the key terms “Patients,” “Doxorubicin,” and “Dose.”

As shown in Figure 4D (Subject terms density plot), “Patients” is the most important Mesh, appearing 557 times. It is followed by “Doxorubicin” (*n* = 328), “Cell” (*n* = 301), “Dose” (*n* = 272), “Tumor” (*n* = 266), “Dox” (*n* = 206), and “risk” (*n* = 188). Among the top 20 Mesh, most are related to the treatment and mechanisms of cardiac toxicity, such as “Cell,” “Tumor,” “Doxorubicin,” “Trastuzumab” “Apoptosis,” “heart failure,” “Nanoparticle” and “Cancer cell.” Some Mesh are associated with experimental modeling, such as “Trial” “vitro” and “Mouse.” This suggests that these fields may represent current research hotspots.

### 3.5 Top research topics in targeted therapy cardiotoxicity

**Figure 5:**
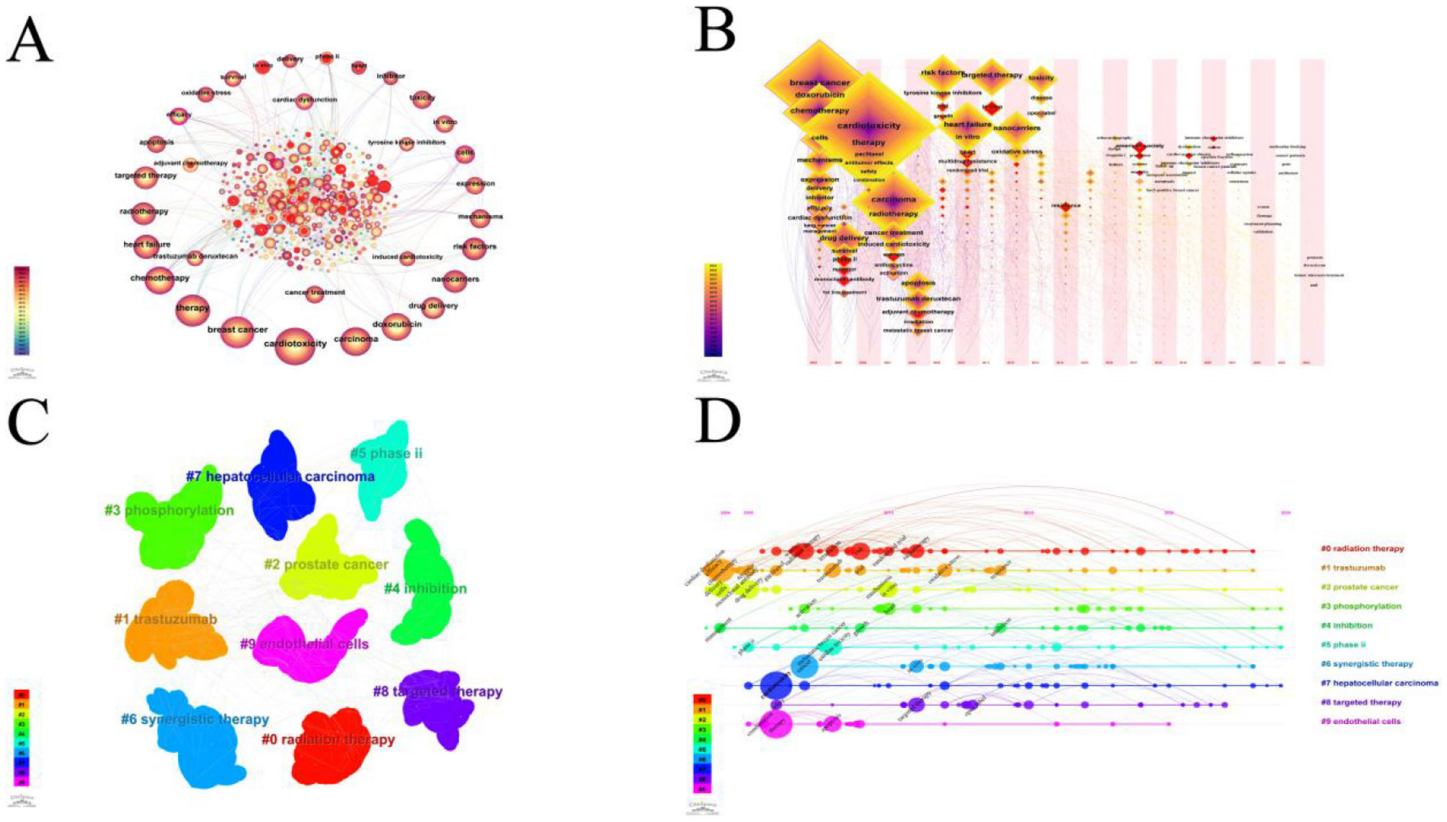
**(A)** A CiteSpace network visualization of keywords related to cardiotoxicity of targeted cancer therapies. Nodes represent keywords and lines representing co-occurrence between keywords connect them. The node size is directly proportional to the keyword frequency. The outermost layer of a node with a purple circle indicates that the keyword it represents has a high centrality. **(B)** The cluster of keywords associated with cardiotoxicity of targeted cancer therapies. Each color represents a different cluster. Each dot represents a unique keyword. The lines between the dots represent two keywords with a co-current relationship. **(C)** The keyword time zone map is associated with the cardiotoxicity of targeted cancer therapies. Keywords are depicted as dots, with the size of each dot corresponding to the frequency of keyword occurrence. Clusters are distinguished by distinct colors, and the links between dots signify co-occurrence of keywords. **(D)** Timeline view of keywords related to cardiotoxicity of targeted cancer therapies. A cluster is depicted as a horizontal line, with the #0 cluster being the largest. The larger the number, the smaller the cluster. The size of the nodes within each cluster reflects the co-citation frequency, and co-citation relationships are shown by the lines between nodes. Each node's occurrence year represents the time it was first co-cited.

**Figure 6:**
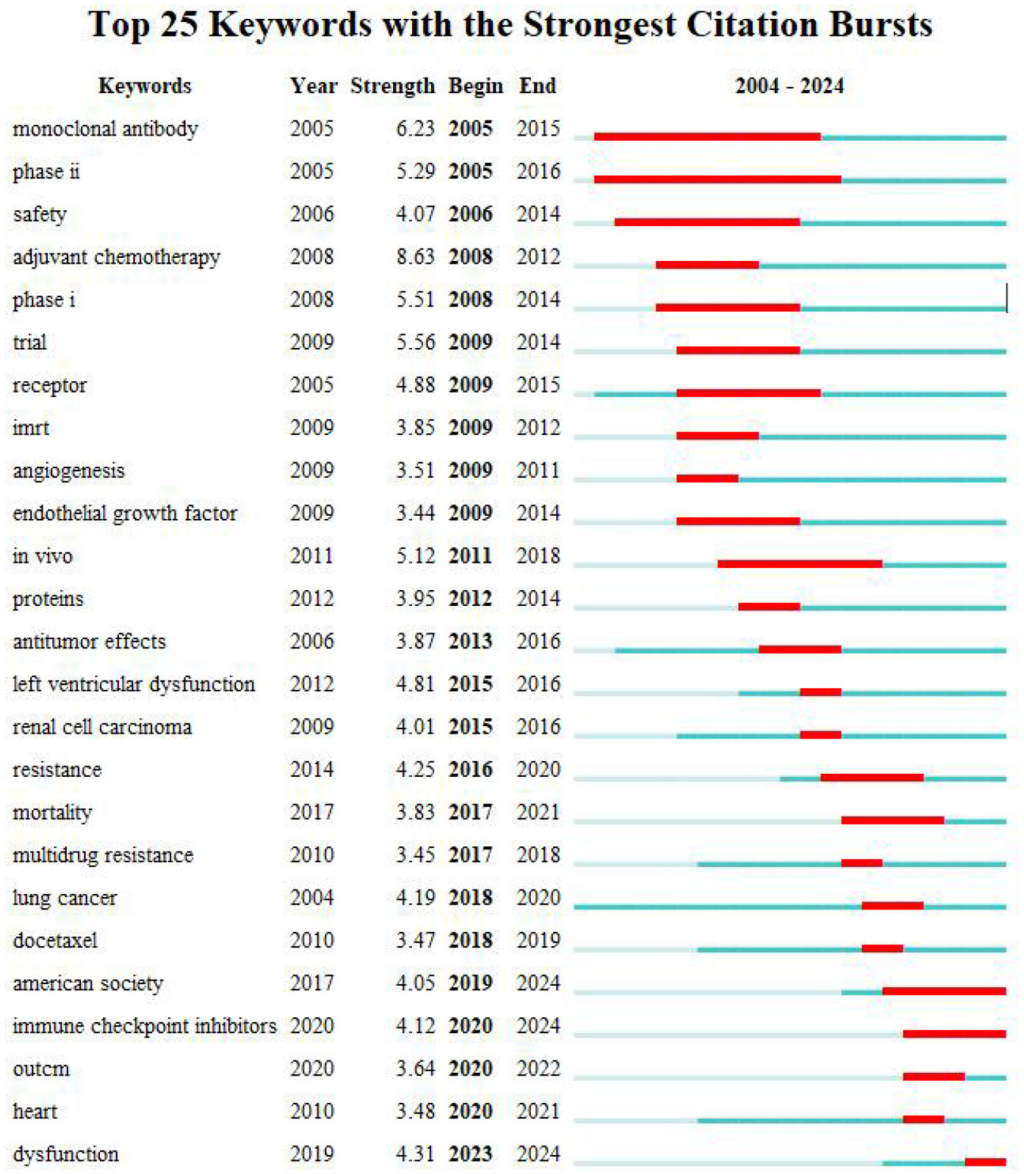
The top 25 keywords exhibit the most pronounced citation bursts. The timeline is depicted as a blue line, with burst periods highlighted in red, indicating the start year, end year, and duration of each burst.

The key terms identified in targeted therapy cardiotoxicity encompassed cardiotoxicity, breast cancer, therapy, carcinoma, chemotherapy, doxorubicin, heart failure, radiotherapy, and risk factors (Figure 5A). To visualize keyword clusters, we generated a network map (Figure 5C), revealing hot spots such as “radiation therapy” (Cluster 0), “trastuzumab” (Cluster 1), “prostate cancer” (Cluster 2), “phosphorylation” (Cluster 3), “inhibition” (Cluster 4), “phase ii” (Cluster 5), “synergistic therapy” (Cluster 6), “hepatocsllulan carcinoma” (Cluster 7), “targeted therapy” (Cluster 8), and “endothelial cells” (Cluster 9). Furthermore, keyword time zone maps (Figure 5B) and timeline views (Figure 5D) were employed to illustrate the evolution of high-frequency keywords. Burst analysis of keywords involved assessing burst intensity and duration, serving as indicators of dynamic fluctuations in keywords over time and offering insights into the development of research pathways (9). Figure 5B presents the high-frequency keywords (*T* > 20). Figure 6 highlights the citation bursts associated with the top 25 keywords. During the early stage (2003–2010), keywords such as “monoclonal antibody,” “phase ii,” “safety,” “adjuvant chemotherapy,” “phase i,” “trial,” “receptor,” “imrt,” “angiogeneis,” and “endothelial growth factor” were prominent. In the subsequent period (2011–2018), “*in vivo*,” “proteins,” “antitumor effects,” “left ventricular dysfunction,” “renal cell carcinoma” “resistance,” “mortality,” “multidrug resistance,” and “lung cancer,” emerged as research hotspots. In recent years (2019–2024), attention has shifted to “docetaxel,” “American society,” “immune checkpoint inhibitors,” “outcm,” “heart,” and “dysfunction.” Additionally, we identified the top five targeted therapy approaches based on the number of citations, as detailed in Table 2. In this table, Degree centrality denotes the number of connections a node has with other nodes in a network. A higher degree in a network indicates higher centrality, suggesting greater importance of the node in the network.

**Table 2:** Top five targeted therapies in the publications.

**Rank**	**Targeted therapy**	**Number of citations**	**Degree**	**Centrality**
1	Trastuzumab	58	14	0.08
2	Tyrosine kinase inhibitor	34	6	0.04
3	Monoclonal antibody therapy	27	15	0.14
4	Immune checkpoint inhibitors	13	6	0.03
5	Pertuzumab	7	1	0

## 4 Discussion

### 4.1 General information

In this study, we focused on cardiotoxicity induced by targeted cancer therapies, encompassing: (1) cardiotoxicity from targeted monotherapies (e.g., trastuzumab, tyrosine kinase inhibitors); (2) synergistic or additive toxic effects arising from combined regimens of targeted agents with conventional therapies (e.g., chemotherapy or radiotherapy). Studies exclusively examining cardiotoxicity of conventional chemo-/radiotherapy without targeted therapy co-administration were deliberately excluded. To systematically map this research landscape, we retrieved relevant literature published in the WOS database between 2004 and 2024. A total of 1,054 articles were subjected to bibliometric visualization analysis. The number of publications on this topic has increased annually since 2004, reflecting the growing attention from the oncology community toward this critical issue.

The remarkable contributions of both China and the United States to academic advancement in this field are underscored by the fact that the top 10 most productive institutions hail from these two countries. This also underscores the strong presence and influence of both nations in this domain. Notably, Shanghai Jiao Tong University ranked third globally in productivity but had the lowest average citation among the top 10 institutions, an intriguing observation. Among these top institutions, MD Anderson, particularly the University of Texas MD Anderson Cancer Center, garnered the highest total citations in 1948, while Massachusetts General Hospital (MGH) boasted the highest average citations at 101.73. This highlights the pivotal roles played by MD Anderson and MGH in pushing the field forward through the publication of high-quality articles that significantly contributed to its advancement. The most cited work at the University of Texas MD Anderson Cancer Center is a review outlining the preventive mechanisms of cardiotoxicity induced by anthraquinone compounds, elucidating the latest molecular mechanisms and offering valuable insights for future research. Concurrently, this institution has extensively explored the cardiotoxic effects of cancer treatments to enhance preventive and treatment strategies. Massachusetts General Hospital (MGH) has developed a risk stratification tool for clinical use, enabling patients to assess their risk before undergoing cancer treatment, thereby alerting them to potential risks of heart failure or other severe cardiovascular toxicities. This tool enhances patient safety significantly. The bilateral dominance of China and the U.S. in both publication volume and citation impact underscores their indispensable roles in shaping global research agendas. To foster the further development of this field, it is imperative to prioritize inter-institutional collaboration and establish more platforms to facilitate cooperation among institutions, which can foster innovative solutions to address unmet challenges in cardiotoxicity management.

The research findings reveal the participation of 8,052 authors in international journal publications. Within the top 10 most active authors, each has authored a minimum of five articles. In the top 10 most cited authors, these individuals have been referenced at least 47 times, signifying their significant contributions to the realm of cardiac toxicity in targeted molecular therapy for tumors. De Lorenzo, Claudia, Abnous, Khalil, and Suter, Thomas M., emerge as the top three authors with the highest number of published papers. Their extensive research achievements have made them key figures in driving this research field, promoting mechanistic insights into cardiotoxicity and the development of new intervention strategies. Thus, to remain abreast of the latest advancements in this field, it is imperative to focus more closely on their work and accord it a higher priority, as their contributions serve as cornerstones for both current and future investigations.

Among the top 10 most prolific journals, the International Journal of Radiation Oncology, Biology, and Physics led the field with the greatest publication output, totaling 20 articles. Following closely was the Journal of Controlled Release and Cancer, each with 19 and 17 articles, respectively, along with the International Journal of Nanomedicine also publishing 16 papers. As depicted in Figure 3A, co-cited journals in molecular biology and genetics primarily received citations from journals in the fields of molecular medicine, medical sciences, and clinical research. This trend indicates a dual focus in the study of cardiotoxicity associated with targeted cancer therapies: basic research and the translation of findings from basic science to clinical applications. The journal “4 Molecular, Biology, Immunology” extensively references the publication “8 Molecular, Biology, and Genetics,” illustrating interdisciplinary collaboration and knowledge sharing aimed at advancing the comprehension and management of cardiac toxicity. Moreover, the majority of the top 10 journals (70%) and co-cited journals (90%) within the Journal Citation Reports first quartile (Q1) division were predominantly high-impact factor (IF) publications. These journals exhibit a dedicated commitment to investigating the cardiotoxic effects of tumor-targeted therapies, thereby significantly enriching this field of study.

The most highly cited article among the top 10 is “Prevention and Monitoring of Cardiac Dysfunction in Survivors of Adult Cancers: American Society of Clinical Oncology Clinical Practice Guideline,” authored by Armenian et al. in 2017. This research offers guidelines for preventing and monitoring cardiac dysfunction in adult cancer survivors, emphasizing the importance of routine cardiac imaging surveillance for specific high-risk survivor groups to enable timely intervention and the prevention or reversal of cardiac dysfunction progression (10). The article “Molecular Mechanisms of Cardiotoxicity of Tyrosine Kinase Inhibition,” authored by Force et al. in 2007, critically examines the cardiac toxicity associated with tyrosine kinase inhibition. It underscores the importance of fostering close collaboration between oncology and cardiology, a perspective that has garnered significant attention as evidenced by its multiple citations (11). In a randomized controlled trial conducted by Gulati et al. in 2016, it was observed that candesartan led to a reduction in left ventricular ejection fraction (LVEF) in early breast cancer patients undergoing adjuvant chemotherapy, while metoprolol did not yield a substantial impact on LVEF decline (12). Candesartan, when used as adjunctive therapy, demonstrated a protective effect against the early onset of overall left ventricular dysfunction, suggesting a potential therapeutic approach to mitigate chemotherapy-induced cardiac functional deterioration (12). These influential studies collectively showcase the current research landscape concerning the impact of molecular targeted therapy on cardiac toxicity, enriching our comprehension of the evolving knowledge framework within this domain.

In bibliometric analysis, co-occurrence analysis of keywords can reveal the central themes of literature (13). Over the last two decades, the realm of cardiotoxicity in tumor-targeted therapy has showcased various significant themes, as illuminated by keyword co-occurrence network analysis.

These themes encompass cardiotoxicity, breast cancer, therapy, carcinoma, chemotherapy, heart failure, doxorubicin, radiotherapy, and risk factors.

The visualization of timelines, keyword time zone maps, and keyword burst analysis offers valuable insights into the evolution of research frontiers in the field. By examining the citation bursts of keywords, we can pinpoint three distinct stages in the evolution of keywords. The initial stage (2003–2010) featured keywords like “monoclonal antibody,” “phase ii,” “safety,” “adjuvant chemotherapy,” “phase i,” “trial,” “receptor,” “imrt,” “angiogeneis,” and “endothelial growth factor” indicating the gradual rise of tumor-targeted therapy during this period (14). Studies have demonstrated the efficacy of trastuzumab as a monotherapy while underscoring the importance of addressing its cardiotoxicity (15). Clinical trials on monoclonal antibodies have been conducted, with other targeted drugs also gaining prominence (16–18). In the middle period (2011–2018) “*in vivo*,” “proteins,” “antitumor effects,” “left ventricular dysfunction,” “renal cell carcinoma” “resistance,” “mortality,” “multidrug resistance,” and “lung cancer,” added to research hotspots. Research on small-molecule tyrosine kinase inhibitors has developed rapidly (19, 20). Resistance to chemotherapy is a major barrier to successful treatment of cancer (21). Chemotherapy research during this time has prioritized overcoming multidrug resistance (MDR) in cancer cells (22). Nanotechnology-based studies have led to the development of innovative smart nanomaterials and effective strategies for combatting MDR, presenting new avenues for cancer treatment. Protein-based nanomedicine, known for its high biocompatibility and low toxicity, emerged as an ideal drug delivery platform for achieving safe and superior therapeutic effects (23). In recent years (2019–2024), “docetaxel,” “american society,” “immune checkpoint inhibitors,” “outcm,” “heart,” and “dysfunction” have become the focus of attention of current research. As depicted in Figures 5C, 6, recent research has primarily concentrated on two main areas of interest. On the one hand, the development of novel nano-delivery vectors to enhance efficacy and mitigate cardiotoxicity (24–27); and the exploration of mechanisms underlying targeted therapy drug-induced cardiac injury, including the use of drugs to block associated mechanisms or employing alternative technologies to prevent, reduce, or treat cardiac injury (28–32).

### 4.2 Research agenda for future work

Our study reveals two critical potential research gaps requiring urgent attention. First, despite the rising clinical use of immune checkpoint inhibitors (ICIs), the cardiotoxicity mechanisms (e.g., ferroptosis, NLRP3 inflammasome activation) remain insufficiently understood and warrant further elucidation. To address this gap, future studies should employ multi-omics approaches to systematically map adverse cardiac trajectories induced by ICIs in combination therapy scenarios. Second, while nanocarriers demonstrate preclinical promise for mitigating cardiotoxicity, established clinical standardization frameworks remain lacking. We recommend developing clinical-grade dual-targeting nanocarriers validated in large-animal models.

The United States and China maintain prominent positions in this field, with the top 10 most productive institutions originating from these nations. Enhanced collaboration among these institutions can significantly advance the future progress of this field. Research on the cardiac toxicity of targeted cancer therapies is rapidly evolving through global cooperation. Analysis of subject terms and keywords reveals two primary areas of focus and future exploration. Firstly, the development of innovative nanocarriers to improve efficacy and reduce cardiac toxicity. Secondly, investigations into the mechanisms of cardiac injury induced by targeted therapeutic agents and the exploration of drug interventions to block these mechanisms or the utilization of alternative techniques for the prevention, alleviation, or treatment of cardiac injury.

Various Western and Chinese medicines have demonstrated potential in preventing and managing cardiovascular toxicity resulting from anticancer treatments. Recent attention has shifted toward the utilization of natural remedies for addressing cardiovascular toxicity associated with anticancer therapies. Studies have highlighted the protective effects of adiponectin, curcumin, resveratrol, *Schisandra chinensis* B, and other novel phytochemicals against doxorubicin-induced cardiac toxicity, leading to reduced mortality rates in cancer patients (33). The consideration of natural remedies in preventing and managing cardiac toxicity induced by targeted cancer therapies is warranted.

Future endeavors should prioritize bolstering preclinical basic and translational research, identifying new targets, and delineating optimal treatment strategies. Collaboration among experts in cardiology, oncology, pharmacy, nursing, and related disciplines is imperative. Emphasis must be placed on the clinical application of targeted drugs and the enhancement of monitoring, prevention, and treatment protocols for cardiac toxicity events associated with targeted cancer therapies. These concerted efforts aim to enhance the safety and efficacy of targeted cancer therapies.

### 4.3 Implications for stakeholders: clinicians, researchers, and patients

From the perspective of clinicians, the visualization findings of this study provide clinically relevant guidance for managing cancer patients undergoing targeted therapies. High-frequency keywords “breast cancer,” “heart failure,” and “doxorubicin,” together with the most-cited keywords “trastuzumab” and “tyrosine kinase inhibitors” (Table 2), underscore that cardiotoxicity risks in tumor-targeted therapeutic regimens remain a prominent research focus. ASCO clinical practice guidelines (10) indicate that patients receiving targeted agents (e.g., trastuzumab) face potential cardiotoxicity risks, necessitating positive attention to risk stratification, cardiotoxicity prevention (e.g., selecting agents with lower cardiotoxic doses, utilizing cardioprotectants such as dexrazoxane), and standardized monitoring practices (e.g., echocardiography, cardiac biomarker monitoring). Furthermore, cluster analysis of “Cardio-oncology” (#2) (Figure 4B) accentuates the need for structured interdisciplinary collaboration. ASCO guidelines (10) further specify that cancer patients manifesting cardiac dysfunction (symptomatic or asymptomatic) require immediate referral to cardiology or cardio-oncology specialists, with anticancer regimen adjustments involving joint decision-making by oncology and cardiology teams to optimize the balance between cancer control and cardiac risk mitigation. Collectively, these findings mandate rigorous implementation of guideline-concordant risk stratification, cardioprotective strategies, and standardized monitoring during targeted therapies, while fostering robust onco-cardiology collaboration to optimize cancer control and cardiac preservation for sustained patient prognosis.

From the perspective of researchers, studies on the cardiotoxicity of targeted therapy have deepened to the level of gene-immune interaction in mechanism exploration. In terms of technological innovation, nano-delivery systems and organoid models have become breakthrough directions. Clinical translation faces challenges such as the popularization of biomarkers and interdisciplinary collaboration. In the future, it is necessary to focus on developing new nanocarriers, exploring targeted therapeutic drugs, screening multi-target components of natural products, and other directions to achieve a balance between efficacy and safety.

From the patient's perspective, the management of cardiotoxicity and the need to improve quality of life during cancer treatment are central to various studies. Although some drugs can improve survival, they may cause deterioration of cardiac function in patients. In reality, patients often face the challenge of balancing treatment and cardiac monitoring, with some young patients even willing to sacrifice part of their healthy lifespan to avoid medication. Patients' varying perceptions of toxicity risks significantly influence their decision-making: young patients are concerned about the impact of drugs during their childbearing years, while elderly patients pay more attention to symptom control. These practical needs highlight the importance of patient-centered research, balancing efficacy and quality of life, and optimizing management pathways through multidisciplinary collaboration and precise monitoring. In the future, it is necessary to incorporate patient-reported outcomes, build a more comprehensive evaluation system, and address the needs of special populations to enhance the safety of treatment and patients' quality of life.

## 5 Limitations

The limitations of this study include the following aspects. Initially, we only selected publications indexed in the WOSCC database, which caused the omission of articles from other sources. Additionally, we only obtained literature published between 2003 and 2024, and earlier literature was not included. Lastly, this study only included English articles, possibly leading to source bias.

## 6 Summary

In summary, we reviewed and analyzed a total of 1,054 papers from 71 countries/regions, 2,058 research institutions, and 8,052 authors. The publication volume concerning cardiac toxicity induced by targeted cancer therapy has exhibited a consistent upward trajectory over the past two decades, indicating a growing interest in this area for the future. The United States and China emerged as the most cited countries, with the University of Texas MD Anderson Cancer Center and Memorial Sloan Kettering Cancer Center as key institutions; Claudia de Lorenzo was the most prolific author, with the greatest citation count. The International Journal of Radiation Oncology, Biology, and Physics had the highest publication output, while the Journal of Clinical Oncology garnered the most co-citations. The most highly cited article was authored by Armenian et al. in 2017, underscoring clinical guidelines' critical role. Analysis of keywords and subject terms revealed prevalent terms in this research domain such as “breast cancer,” “chemotherapy,” “doxorubicin,” “drug delivery,” and “nanoparticles.” This analysis identified two critical research gaps requiring urgent attention: the insufficient understanding of cardiotoxicity mechanisms (e.g., ferroptosis, NLRP3 inflammasome activation) associated with ICIs, and the lack of established clinical standardization frameworks for promising preclinical nanocarrier strategies. Recent research trends and future directions primarily focus on two key areas: the development of novel nanocarriers aims to enhance therapeutic efficacy while reducing cardiac toxicity, and the exploration of mechanisms underlying cardiac injury caused by targeted therapeutic drugs is crucial, along with the investigation of drug interventions to counter these mechanisms or the application of alternative techniques for the prevention, alleviation, or treatment of cardiac injury. Clinically, integrating cardiotoxicity surveillance protocols and promoting interdisciplinary collaboration between oncology and cardiology are essential. Researchers should prioritize translational studies on nanomedicine, explore molecular mechanisms, and evaluate natural compounds for cardioprotection in the future. Policymakers should fund cross-institutional global initiatives to standardize toxicity assessment metrics and foster multidisciplinary research frameworks, enhancing the safety and efficacy of targeted cancer therapies.
